# Puerarin Mitigates Diabetic Hepatic Steatosis and Fibrosis by Inhibiting TGF-*β* Signaling Pathway Activation in Type 2 Diabetic Rats

**DOI:** 10.1155/2018/4545321

**Published:** 2018-07-02

**Authors:** Biyu Hou, Yuerong Zhao, Guifen Qiang, Xiuying Yang, Chunyang Xu, Xi Chen, Chenge Liu, Xiaobo Wang, Li Zhang, Guanhua Du

**Affiliations:** ^1^State Key Laboratory of Bioactive Substance and Function of Natural Medicines, Institute of Materia Medica, Chinese Academy of Medical Sciences, and Peking Union Medical College, Beijing 100050, China; ^2^Beijing Key Laboratory of Drug Target Identification and Drug Screening, Institute of Materia Medica, Chinese Academy of Medical Sciences, & Peking Union Medical College, Beijing 100050, China; ^3^School of Traditional Chinese Medicine, Guangdong Pharmaceutical University, Guangzhou 510006, China; ^4^College of Pharmacy, Harbin University of Commerce, Harbin 510006, China

## Abstract

Lipid metabolism disorder and inflammation are essential promoters in pathogenesis of liver injury in type 2 diabetes. Puerarin (PUR) has been reported to exert beneficial effects on many diabetic cardiovascular diseases and chemical-induced liver injuries, but its effects on diabetic liver injury and its mechanism are still unclear. The current study was designed to explore the therapeutic effect and mechanism of PUR on liver injury in a type 2 diabetic rat model induced by a high-fat diet combined with low-dose streptozotocin. The diabetic rats were treated with or without PUR (100 mg/kg/day) by gavaging for 8 weeks, and biochemical and histological changes in liver were examined. Results showed that treatment with PUR significantly attenuated hepatic steatosis by regulating blood glucose and ameliorating lipid metabolism disorder. Liver fibrosis was relieved by PUR treatment. PUR inhibited oxidative stress and inflammation which was associated with inactivation of NF-*κ*B signaling, thereby blocking the upregulation of proinflammatory cytokines (IL-1*β*, TNF-*α*) and chemokine (MCP-1). This protection of PUR on diabetic liver injury is possibly related with inhibition on TGF-*β*/Smad signaling. In conclusion, the present study provides evidence that PUR attenuated type 2 diabetic liver injury by inhibiting NF-*κ*B-driven liver inflammation and the TGF-*β*/Smad signaling pathway.

## 1. Introduction

Diabetes mellitus (DM) occupies a leading position in the morbidity and mortality statistics, especially in developed countries [1]. Other than chronic macrovascular and microvascular complications, DM is also associated with liver-related mortality and has become an increasing risk of hepatocellular carcinoma [2]. Growing evidence suggests that patients with T2DM are at a particularly high risk for nonalcoholic fatty liver disease (NAFLD) [3]. Moreover, diabetes is an independent risk factor for NAFLD progression, hepatocellular carcinoma development, and liver-related mortality [4].

The progressive lipid deposition and fibrosis in liver is the typical character of NAFLD [5]. Apart from insulin resistance and hyperinsulinemia, lipid metabolism disorder in liver and subsequently increased oxidative stress accompanied with inflammation contribute to the progression of T2DM along with NAFLD and lead to fibrosis and potentially cirrhosis [6]. Activation of hepatic stellate cells into contractile, proliferative, and fibrogenic cells in liver injury is a dominant link in the process of liver fibrosis [7]. The TGF-*β*/Smad signaling pathway plays a major role in the activation of HSCs in liver fibrosis.

Currently, weight control and lifestyle modification are considered as first-line therapy [8]. Pharmacological treatment including insulin-sensitizing agents [9], antioxidants [10], and metformin [11] may be effective in ameliorating NAFLD and preventing hepatic fibrosis. Puerarin (daidzein-8-C-glucoside) (PUR) is the most important phytoestrogen extracted from Chinese medicinal herb Gegen, the root of the wild leguminous creeper *Pueraria lobata* (kudzu root). Previous pharmacological researches have demonstrated that PUR possessed a series of beneficial activities on hangover [12], osteoporosis [13], neurological dysfunction [14], and fever [15], in clinical treatment and experimental research. However, emerging data has shown that PUR also has a beneficial effect on diabetes mellitus and its complication such as cardiovascular diseases [16] and diabetic nephropathy [17]. Interestingly, PUR has been certified to ameliorate acute alcoholic liver injury [18] and exert protection against chemical injuries including CCL_4_ [19], lead [20], and alcohol [21]. However, its effects on liver injury during T2DM and the underlying mechanism are unclear. In the present study, we investigated the effects of PUR on diabetic liver in a high-fat diet combined with low-dose streptozotocin- (STZ-) induced type 2 diabetic rats. Our results suggested that PUR prevented the pathological progression of hepatic fibrosis by reducing oxidative stress and anti-inflammatory effects via inhibition of NF-*κ*B signaling and the TGF-*β*/Smad signaling pathway.

## 2. Materials and Methods

### 2.1. Animals and Treatments

Male Sprague-Dawley (SD) rats (150–170 g) were obtained from Beijing HFK Bioscience Co. Ltd (Beijing, China). Animals were raised in a SPF environment (22–25°C, humidity 60–70%, 12 h light/12 h dark cycle). All animal experiments conformed to guidelines established by the animal care committee of the Institute of Materia Medica, Chinese Academy of Medical Sciences. After one week of acclimatization, a rat model of liver fibrosis with type 2 diabetes was induced as previously described with little modification [22]. Briefly, rats were fed with a high-fat and high-sucrose diet (standard diet supplemented with 10% sucrose, 10% lard stearin, 2% cholesterin, and 0.5% cholic acid) for 4 weeks. 35 mg/kg STZ (Sigma-Aldrich, St. Louis, MO) was injected intraperitoneally in 0.1 M citrate buffer (pH 4.4) after an overnight fasting, while normal animals received only citrate buffer. Rats with fasting blood glucose (FBG) higher than 10 mM and lower than 25 mM were considered as diabetic and received a high-fat diet (10% lard stearin, 2% cholesterin, and 0.5% cholic acid) for another 8 weeks. During this time, diabetic rats were randomly assigned into two groups: diabetic model rats (DM) treated with vehicle and diabetic rats with oral administration of PUR at 100 mg/kg/day (PUR). Meanwhile, normal control rats (NC) were also administered with vehicle and received a standard chow diet throughout the experiment.

### 2.2. Blood Collection and Tissue Preparation

At the end of the 8-week treatment, rats were sacrificed after fasting overnight. Blood lipids of triglycerides (TG), total cholesterol (T-CHO), high-density lipoprotein (HDL), low-density lipoprotein (LDL), and hepatic enzyme activities of alanine transaminase (ALT) and aspartate transaminase (AST) were detected with an automatic analyzer (Toshiba Accute TBA-40FR, Toshiba Corporation, Tokyo, Japan) for the liver function test using a common commercial kit (BioSino Bio-Technology and Science, Beijing, China). The activity of hepatic lipase was assayed following the instruction of the commercial kit (Jiancheng Biotech Co., Ltd., Nanjing, China). Total antioxidant capacity of liver tissue was assessed with ABTS Kit, and the activity of catalase in the liver was tested according to the protocol of the manufacturer (Beyotime Biotech, China). Briefly, after mixing the liver tissue of rat with excessive H_2_O_2_ for 3 min, the remaining H_2_O_2_ was detected, and the activity of CAT was determined as the amount of H_2_O_2_ converted into water and oxygen per min. The specific markers for oxidative stress including malondialdehyde (MDA) and superoxide dismutase (SOD) of serum were analyzed by commercial kits (Jiancheng Biotech Co., Ltd., Nanjing, China). The serum level of 8-OHdG is assessed according to the manufacturer's instructions using a commercially available kit (Cusabio, Wuhan, China). Liver specimens were fixed in 10% (*w*/*v*) neutral formaldehyde, then dehydrated with a graded-series of alcohol and embedded in paraffin wax for histological examinations.

### 2.3. Histopathology and Immunohistochemistry Morphological Analysis

Paraffin sections (4 *μ*m thick) of the fixed and processed livers were stained with hematoxylin–eosin (HE) reagent, Masson trichrome reagent, and Oil Red O staining reagent following the standard steps in the instructions. The other paraffin-fixed liver specimens were sliced into 6 *μ*m thick sections and stained with picric acid–Sirius red (0.1% Sirius red in saturated aqueous picric acid) (Sigma, USA) to detect hepatic fibrosis, mainly collagen types I and III. The slices were imaged under a microscope (Nikon Eclipse Ti-U, Nikon Corporation, Tokyo, Japan). Five randomly selected images per section were digitally captured, and the optical density of positive area was analyzed using ImageJ (NIH, Bethesda, MD) software.

### 2.4. Immunohistochemical Staining of *α*-SMA, TGF-*β*1, CD68, and 3-Nitrotyrosine

The sections were dewaxed in xylene and dehydrated in alcohol. Antigen retrieval was achieved by microwaving in citric saline at 95°C for 3 min, then sections were treated with 3% hydrogen peroxide for 25 min. After blocking in 5% BSA, sections were incubated at 4°C overnight with primary antibody against rabbit anti-*α* smooth muscle actin (*α*-SMA) antibody or rabbit antitransforming growth factor *β*1 (TGF-*β*1) (1 : 1000; CST, USA), CD68 antibody (1 : 100; CST, USA), and 3-nitrotyrosine (3-NT) antibody (1 : 500; Santa Cruz). Then, sections were incubated with HRP-conjugated goat anti-rabbit IgG (Dako, Wuhan, China) for 50 min at room temperature. *α*-SMA, TGF-*β*1, and CD68 expressions were visualized by DAB (Dako, Wuhan, China) staining. Sections were then examined under an optical microscope (Nikon Eclipse Ti-U, Nikon Corporation, Tokyo, Japan). Five randomly selected images were digitally captured (400x magnification), and the optical density was quantified by ImageJ (NIH, Bethesda, MD) software.

### 2.5. Quantitative Real-Time PCR

Total RNA was extracted using TRIzol (Life Technologies, Grand Island, NY), followed by reverse transcription of total RNA to cDNA. cDNA was synthesized using a high-capacity cDNA reverse transcription kit (Applied Biosystems, Foster city, CA). cDNA subsequently underwent quantitative real-time polymerase chain reaction (PCR) using the CFX96™ real-time system (Bio-Rad, Singapore). The sequences of the primers are provided in Table 1.

### 2.6. Western Blot

Liver tissue was homogenized in cold RIPA buffer containing proteases and phosphatase inhibitors. Lysates were subjected to SDS-PAGE and then transferred onto the PVDF membrane (Millipore, Billerica, MA, USA). After blocking, immunoblotting was incubated with the primary antibodies: rabbit GAPDH antibody (Santa Cruz, CA) as controls for loading and transfer as well as rabbit phospho-NF-*κ*B (p65) antibody, rabbit NF-*κ*B (p65) antibody, rabbit TGF-*β* antibody, rabbit phospho-Smad2/Smad3 antibody, rabbit Smad2/Smad3 antibody (CST, USA), and rabbit collagen I antibody (Abcam, USA); then goat anti-rabbit HRP (Beijing ComWin Biotech, Beijing, China) was applied and was detected using an enhanced chemiluminescence (ECL) kit (Beijing ComWin Biotech, Beijing, China). Signals were scanned and visualized by ChemiDoc-It® 510 image system (Upland, CA, USA). The ratio of the protein interested was subjected to GAPDH and was densitometrically analyzed by ImageJ software (NIH, Bethesda, MD).

### 2.7. Statistical Analysis

All data are expressed as mean ± SEM. One-way analysis of variance (ANOVA) with a post hoc Dunnett test was used to determine statistically significant differences among the three groups. A value of *p* < 0.05 was considered statistically significant.

## 3. Results

### 3.1. Puerarin Ameliorated the General Parameters of Diabetic Rats

**Figure 1:**
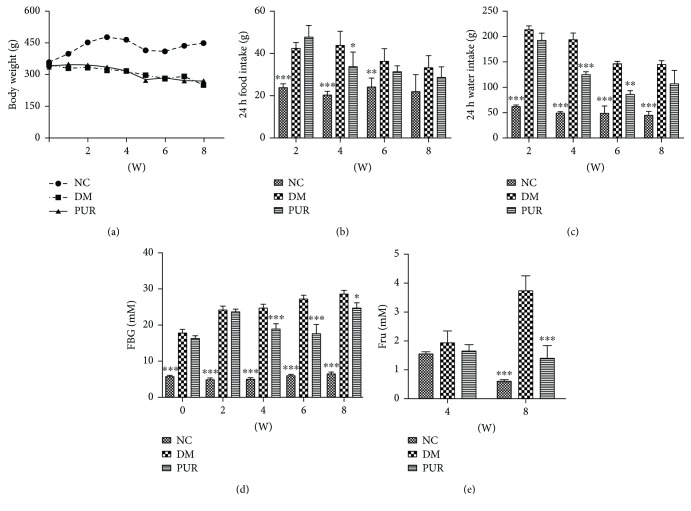
PUR ameliorated the metabolic parameters of diabetic rats. PUR 100 mg/kg was orally administered to diabetic rats for eight weeks, and the general parameters of diabetic were assessed. (a) Body weight, (b) 24 h food intake, (c) 24 h water intake, (d) fasting blood glucose, and (e) serum level of fructosamine. Data are presented as mean ± SEM. *n* = 8 per group, ^∗^*p* < 0.05, ^∗∗^*p* < 0.01, and ^∗∗∗^*p* < 0.001, compared versus DM.

Throughout the 8-week administration, diabetic rats showed a significant decrease in body weight with increased food and water intake compared with normal rats. PUR treatment moderated the body weight and mildly ameliorated polydipsia and polyphagia of diabetic rats (Figures 1(a)–1(c)). The constantly growing high level of blood glucose was decreased by PUR treatment with statistical significance (Figure 1(d)). This amelioration on blood glucose is certified by the fructosamine result which indicated the 2- or 3-week blood glucose level (Figure 1(e)).

### 3.2. Puerarin Improved Deteriorated Liver Function and Structure of Diabetic Rats

**Figure 2:**
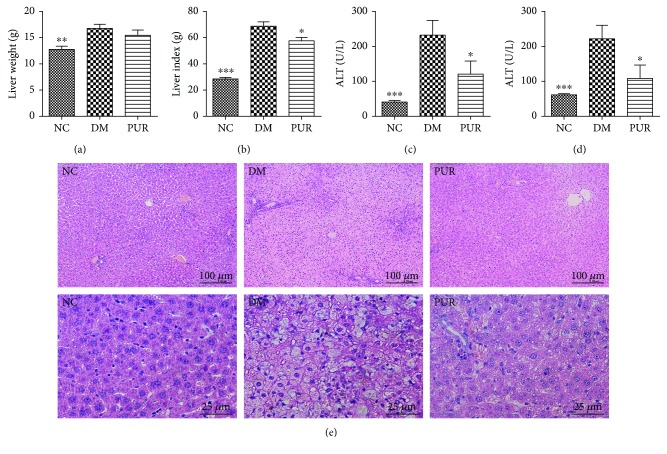
PUR attenuated diabetic liver morphological and functional deterioration. PUR 100 mg/kg was orally administered to diabetic rats for eight weeks, and the liver weight (a), liver coefficient (b), and serum level of ALT (c) and AST (d) were determined. (e) Representative photomicrographs of HE staining, magnified 100x and 400x. Data are presented as mean ± SEM. *n* = 8 per group, ^∗^*p* < 0.05, ^∗∗^*p* < 0.01, and ^∗∗∗^*p* < 0.001 compared versus DM.

Compared with the normal group, the diabetic liver exhibited hypertrophy and increase in liver index whereas PUR 100 mg/kg reduced the liver index significantly (Figures 2(a) and 2(b)). Next, liver impairment markers (ALT and AST) were detected to investigate the effect of PUR on diabetic liver injury. The serum concentrations of ALT and AST significantly increased in the DM group compared with the NC group (*p* < 0.001). However, PUR significantly decreased the levels of both markers in the serum compared with the DM group (*p* < 0.05) (Figures 2(c) and 2(d)). HE staining results (Figure 2(e)) indicated that PUR had an obvious amelioration on liver morphological change. Compared with the NC group, the DM group showed severe hepatic lobule damage, and a periportal and interstitial fibrous connective tissue increase was observed. A lot of fatty degeneration of hepatic cells happened around the central veins. Treatment with PUR significantly moderated this morphological change against diabetic hepatic steatosis, since the liver of the PUR group had a much more complete structure of hepatic lobules, with its liver cells more organized, and there was less inflammatory cell infiltration.

### 3.3. Puerarin Regulated Lipid Metabolism Disorder and Lipid Deposition in Liver of Diabetic Rat

**Figure 3:**
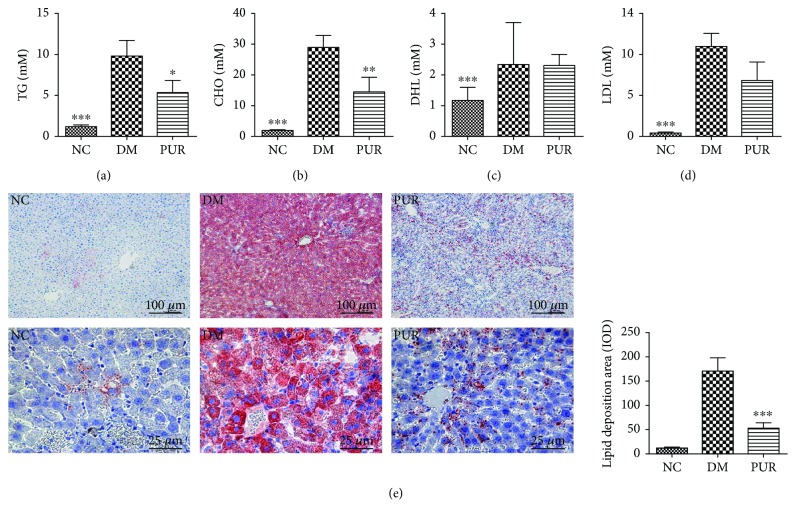
PUR improved lipid metabolism and hindered lipid deposition in the diabetic liver. PUR 100 mg/kg was orally administered to diabetic rats for eight weeks, and the serum levels of TG (a), TCHO (b), HDL (c), and LDL (d) were measured. (e) Representative picture of lipid accumulation in liver by Oil Red O staining magnified by 100x and 400x, and the quantitative results of Oil Red O staining positive area assessed by ImageJ. Data are presented as mean ± SEM. *n* = 8 per group, ^∗^*p* < 0.05, ^∗∗^*p* < 0.01, and ^∗∗∗^*p* < 0.001 compared versus DM.

As shown in Figures 3(a)to 3(d), a marked high level of serum TG, TCHO, and LDL was observed in diabetic rat compared with the normal group, while treatment of PUR effectively ameliorated the lipid metabolism disorder in diabetic rats. We further examined the effect of PUR on liver lipid accumulation by Oil Red O staining. As exhibited in Figure 3(e), there was large amount of deposition of lipids in diabetic liver. A great deal of steatosis hepatocytes showed speckled distribution around the central vein with a heavier lesion. PUR largely changed this lipid distribution, and there was milder steatosis hepatocyte gathered around the central veins.

### 3.4. Puerarin Attenuated Liver Fibrosis

**Figure 4:**
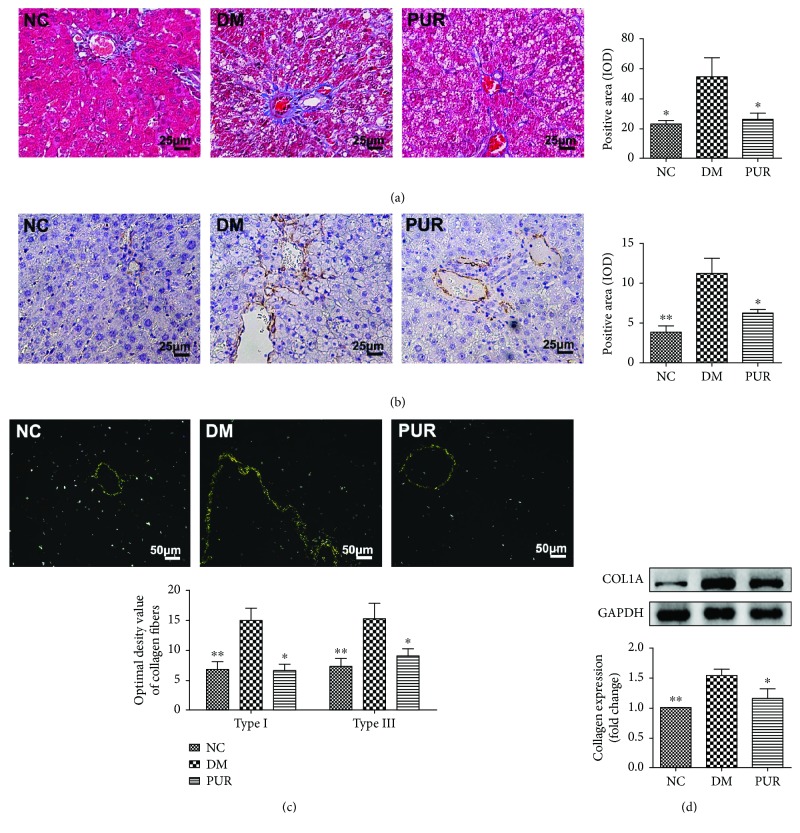
PUR hindered diabetic liver fibrosis and excessive ECM production. (a) Representative picture of liver fibrosis by Masson staining magnified by 400x, and the quantitative results of positive area assessed by ImageJ. (b) Immunohistochemical staining of *α*-SMA, magnified by 400x, and the quantitative results of *α*-SMA staining. (c) Picric acid-Sirius red staining of liver collagen fibers, magnified by 100x and 200x, and the quantitative results of type I and III collagens. Data are mean ± SEM, *n* = 5 per group. (d) Western blot analysis for COL1A1 expression in the liver. Data are mean ± SEM. *n* = 6 per group, replicated twice. ^∗^*p* < 0.05 and ^∗∗^*p* < 0.01 compared versus DM.

In diabetic rats, lipid metabolism disorder and constantly high-level glucose cause liver dysfunction, subsequently resulting in the development of liver fibrosis. We next focused on the therapeutic effect of PUR on liver fibrosis. As shown in the Masson staining result (Figure 4(a)), diabetic liver displayed obvious connective tissue hyperplasia and increase in the extracellular matrix (ECM) content. Collagen accumulated surrounded the lobules, which resulted in large fibrous septa and pseudolobule formation. However, in the PUR treatment groups, these alterations in the liver sections were remarkably reduced. Immunohistochemical staining of *α*-SMA showed that positive cells of *α*-SMA increased markedly and distributed in the portal area, the fiber interval in the DM group (Figure 4(b)). Compared with the DM group, PUR reduced the expression of *α*-SMA with a significant difference (*p* < 0.05). We further perform picric acid-Sirius red staining and Western blot to confirm the collagen formation in each group. As shown in Figure 4(c), there were significantly higher levels of type I and III collagens in the DM group compared with the NC group, while type I and III collagen levels were largely lessened in diabetic rat livers treated with PUR. Western blot results for collagen 1a1 (COL1A1) also confirmed the results that PUR 100 mg/kg/day hindered excessive ECM production in diabetic liver.

### 3.5. Puerarin Improved Glucose Metabolism and Reduced De Novo Lipogenesis in Diabetic Liver

**Figure 5:**
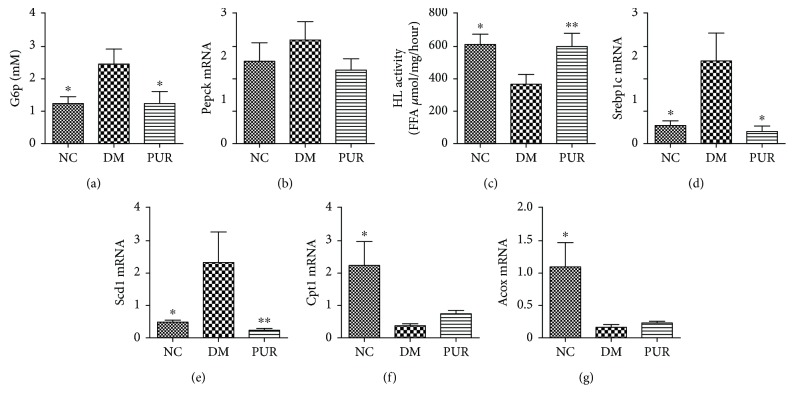
PUR improved glucose metabolism and inhibited lipogenesis in diabetic liver. PUR 100 mg/kg was orally administered to diabetic rats for eight weeks, and mRNA expression in liver tissue of Pepck (a) and G6p (b) was detected by real-time PCR. Hepatic lipase activity was assayed (c), and mRNA expressions in liver tissue of Srebp1c (d), Scd1 (e), Cpt1a (f), and Acox (g) were detected by real-time PCR. Data are presented as mean ± SEM, *n* = 5-8 per group, replicated twice. ^∗^*p* < 0.05 and ^∗∗^*p* < 0.01 compared versus DM.

To elucidate the mechanisms by which PUR effectively ameliorated hepatic steatosis in diabetic rats, we first assayed the gene expression of the key enzyme in gluconeogenesis such as Pck1 and G6p. The results showed that PUR treatment effectively reduced the gluconeogenic gene compared with the diabetic model group (Figures 5(a) and 5(b)). We next measured the activity of hepatic lipase, which hydrolyses circulating TG, VLDL, and CM into glycerin and free fatty acid. The results showed that decreased enzyme activities in diabetic rat liver were restored by PUR treatment (Figure 5(c)). As is shown in Figures 5(d)–5(g), compared with normal rats, the key factor in lipogenic genes including sterol regulatory element-binding protein 1c (Srebp1c) and stearoyl-CoA desaturase 1 (Scd1) were significantly upregulated while the key gene in *β*-oxidation including acyl-CoA oxidase (Acox) and carnitine palmitoyltransferase 1A (Cpt1a) were downregulated. PUR prominently reduced the expression in lipogenic genes, yet had little influence on *β*-oxidation in diabetic liver. These data indicated that PUR prevents hepatic steatosis, effectively improving the glucose metabolism and decreasing de novo lipogenesis in diabetic rats' liver.

### 3.6. Puerarin Suppressed Oxidative Stress in Diabetic Rats

**Figure 6:**
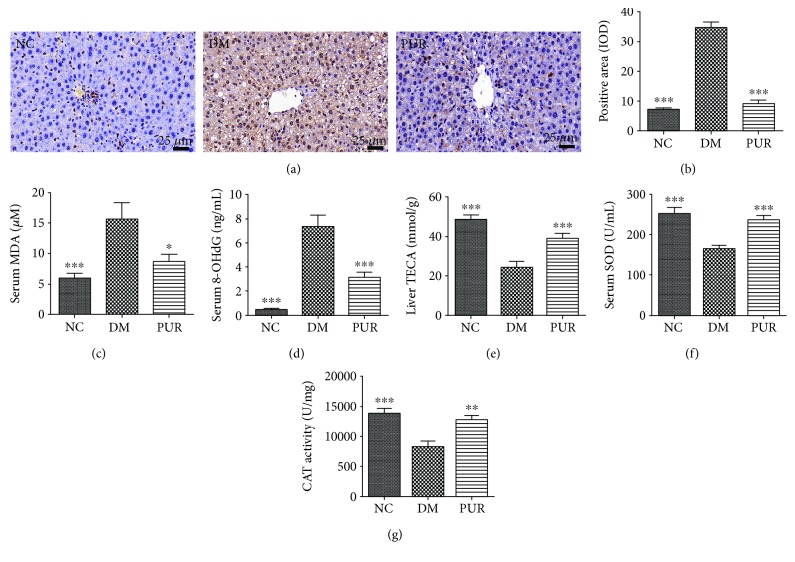
PUR relieved oxidative stress in diabetic rats. PUR 100 mg/kg was orally administered to diabetic rats for eight weeks. (a) Representative picture of immunohistochemical staining of 3-NT, magnified by 400x, and the quantitative results of positive area assessed by ImageJ (b). Data are mean ± SEM, *n* = 5 per group. Serum levels of MDA (c) and 8-OHdG (d) were measured. Total antioxidant capacity of liver tissue was assayed and expressed as Trolox equivalent antioxidant capacity (e), the activity of SOD (f) of serum, and activity of CAT of liver tissue (g) were measured. Data are presented as mean ± SEM, *n* = 8 per group. ^∗^*p* < 0.05, ^∗∗^*p* < 0.01 and ^∗∗∗^*p* < 0.001 compared versus DM.

As shown in Figures 6(a) and 6(b), compared with normal rats, there was a large amount of 3-NT suffused in the liver of diabetic rats. PUR suppressed the expression of 3-NT in liver significantly. Consistent with the result of 3-NT, the serum level of lipid peroxidation end products of MDA in diabetic rats also rose markedly (*p* < 0.001) (Figure 6(c)), while PUR reduced this lipid peroxidation end product accumulation with statistical significance (*p* < 0.05). Moreover, PUR lowered the serum level of 8-OHdG, which is the production of damaged DNA induced by oxidative stress (Figure 6(d)). The total antioxidant capacity was restored by PUR treatment (Figure 6(e)); the activities of key antioxidant enzymes including SOD and liver CAT were also elevated (Figures 6(f) and 6(g)). Taken together, PUR suppressed diabetic rats' oxidative stress.

### 3.7. Puerarin Hinders Inflammation Infiltration in Diabetic Liver

**Figure 7:**
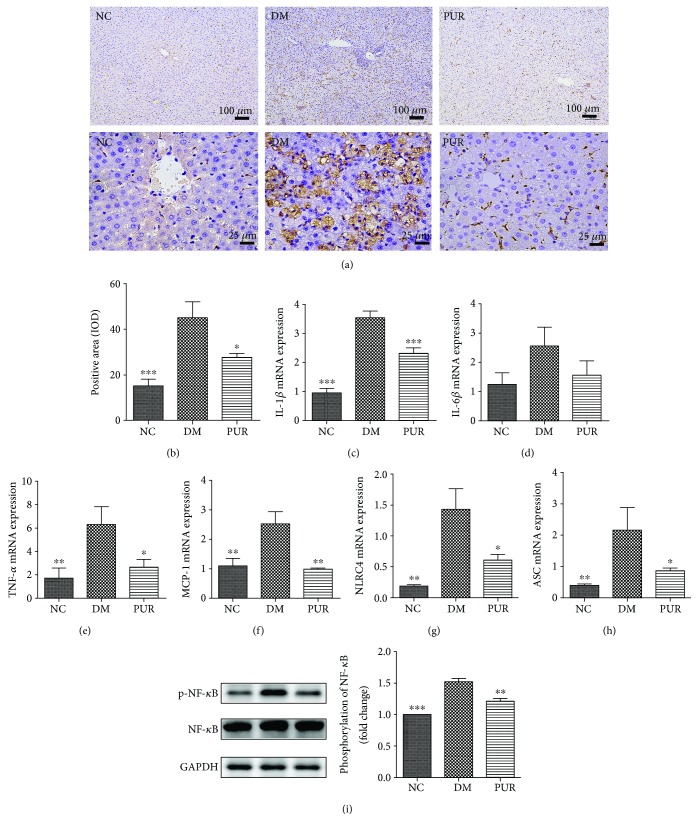
Effect of PUR on inflammation in diabetic liver. (a) Representative picture of liver CD68 staining, magnified by 400x. (b) The quantitative results of CD68 staining assessed by ImageJ. Data are presented as mean ± SEM, *n* = 5 per group. mRNA expressions in liver tissue of IL-1*β* (c), TNF-*α* (d), IL-6 (e), MCP-1 (f), NLRC (g), and and ASC (h) were detected by real-time PCR. (i) Western blot analysis for phosphate NF-*κ*B/p65 in liver tissue. Data are presented as mean ± SEM, *n* = 8 per group, replicated twice. ^∗^*p* < 0.05, ^∗∗^*p* < 0.01, and ^∗∗∗^*p* < 0.001 compared versus DM.

Inflammation plays an important role in the pathogenesis of diabetic hepatic injuries.  Figure 7(a) shows representative photomicrographs of macrophage infiltration in the immunohistochemical staining of CD68. The results of the quantitative analysis of macrophage infiltration (Figure 7(b)) showed that significant macrophage infiltration increased in diabetic rat liver, while PUR hindered this process. To further confirm the effect of PUR on inflammation, tissue inflammatory factors (IL-1*β*, IL-6, TNF-*α*, and MCP-1) were assessed by qPCR. As shown in Figures 7(c)–7(f), PUR reduced the increase of IL-1*β*, IL-6, TNF-*α*, and MCP-1 in diabetic liver. Moreover, PUR also reduced inflammasomes such as NRLC and downregulated the gene expression of apoptosis-associated speck-like protein containing A card (ASC), which is a key adaptor molecule required for inflammatory processes. Western blotting analysis (Figure 7(g)) demonstrated that the NF-*κ*B signaling pathway was highly activated in the diabetic liver as increased phosphorylation of NF-*κ*B/p65 was observed. In contrast, treatment with PUR largely inactivated the pathway in the diabetic liver.

### 3.8. Puerarin Inhibited TGF-*β* Induced Smad2/3 Signaling Pathway Activation

**Figure 8:**
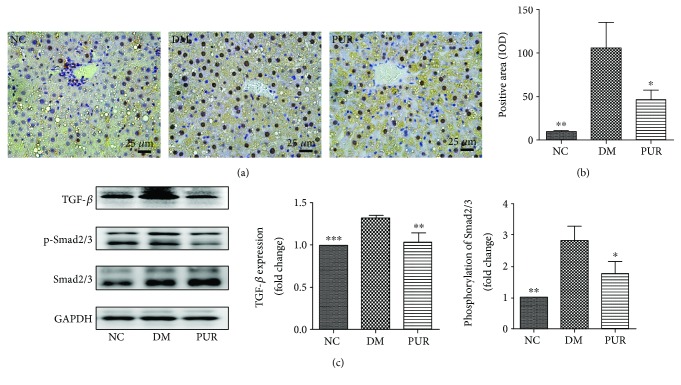
The inhibition of PUR on TGF-*β* induced Smad2/3 signaling pathway activation. (a) Immunohistochemical staining of TGF-*β*, magnified by 400x. (b) Quantitative results of TGF-*β* staining assessed by ImageJ. Data are presented as mean ± SEM, *n* = 5 per group. (c) Western blot analysis for TGF-*β* protein expression and phosphate Smad2/3 in liver tissue. Data are presented as mean ± SEM, *n* = 8 per group, replicated twice. ^∗^*p* < 0.05, ^∗∗^*p* < 0.01, and ^∗∗∗^*p* < 0.001 compared versus DM.

Immunohistochemical staining of TGF-*β* showed that positive cells of TGF-*β* increased markedly and distributed in the portal area, the fiber interval in the DM group (Figure 8(a)). Compared with the DM group, PUR reduced the expression of TGF-*β* with significant difference (*p* < 0.05) (Figure 8(b)). Western blotting analysis (Figure 8(c)) demonstrated that PUR significantly suppressed the TGF-*β*/Smad2/3 signaling pathway which was highly activated in the diabetic liver (*p* < 0.05).

## 4. Discussion

In the present study, we confirmed the therapeutic effect of PUR on diabetic liver injury by regulating glucose and lipid metabolism disorder and alleviating oxidative stress and inflammation in a rat model of type 2 diabetes. Moreover, PUR ameliorated liver fibrosis and reduced the hepatic ECM accumulation associated with suppressing TGF-*β*/Smad signaling.

T2DM patients stand a good chance of developing nonalcoholic fatty liver disease with an incidence rate as high as 50% [23]. To closely mimic the pathogenesis of human disease, we developed the rat model of type 2 diabetes by continuously feeding the diabetic rats with high-fat diet as previously reported [22]. Liver is the central organ in lipogenesis, gluconeogenesis, and cholesterol metabolism. Hepatic steatosis under condition of type 2 diabetes is a result of imbalance in the uptake, synthesis, export, and oxidation of free fatty acids [24]. In the present study, PUR exerted a stable effect on reducing blood glucose throughout eight weeks of experiment. Interestingly, we observed that PUR had a mild influence on the gene expression of the enzyme in gluconeogenesis such as Pepck and G6Pase in the liver. This regulation on blood glucose is confirmed in another study both on high-fat diet-induced and db/db diabetic mice. The underlying mechanisms are involved in activation of GLP-1R to protect *β*-cell survival [25]. To further explain the mechanism of PUR's effect on glucose metabolism, it is necessary to find out whether PUR or its metabolites are accessible to the major metabolizing organs. In Meezan et al.'s research, the accumulation of intact PUR was found in the kidney and liver, which suggested that PUR could be a substrate for SGLT2 in the kidney [26]. This may then suppress renal tubular glucose reabsorption by inhibiting renal SGTL2, thereby increasing urinary glucose excretion. Also, Meezan et al. demonstrated that PUR was also distributed in pancreatic tissue. This indicated that PUR penetrates into the pancreas and may be effective in preventing islet cells from the toxic action of reactive oxygen species in diabetes [27]. These results helped explain the mechanism of PUR on glucose metabolism and amelioration on diabetic complication.

Under type 2 diabetes, insulin resistance causes peripheral adipocytes to undergo lipolysis. Free fatty acids are then released to the bloodstream and eventually accumulate in the liver [28]. In our research, we found that PUR significantly decreased fat accumulated in the liver by Oil O Red staining. The effect of PUR on regulating lipid metabolism disorder is associated with suppression lipogenesis evidenced by the downregulating gene expression of Scd1. Moreover, SREBP-1c has been identified as mediators of the transcriptional effects of insulin and glucose on glycolytic and lipogenic gene expression [29]. In our research, PUR also had suppression on the expression of SREBP-1c. Hepatic lipase is a key enzyme associated with the serum level of TG. Researchers have demonstrated that reduced hepatic lipase will lead to clearance disorder of CM and VLDL and subsequently the accumulation of TG in peripheral blood [30]. Consistent with our result, we found that hepatic lipase activity is significantly reduced, and PUR elevated the deactivated enzyme which might be another mechanism improving lipid metabolism disorder.

Lipid metabolism disorder under diabetes circumstances such as increased TCHO, TG, and LDL and a largely deposited lipid in the liver will subsequently lead to oxidant stress and then trigger the further structural and functional lesion according the famous “two-hit” hypothesis [31]. In this study, consistent with the hyperlipidemia in diabetic liver, we observed that MDA, one of the end products of lipid peroxidation, as well as another biomarker of oxidative DNA damage, 8-OHdG [32], increased correspondingly in diabetic rats. The results indicated that oxidative stress was significantly boosted. In our study, PUR exerted an obvious reduction on liver lipid accumulation. Moreover, it decreased the subsequent MDA collection and prevented further oxidative DNA damage as well as improved the total antioxidant capacity of diabetic liver. PUR also elevated the activities of enzymes defending oxidative stress including SOD and CAT. However, PUR, as an isoflavone glycoside, has been reported in many studies that it has good antioxidant effects; the reduction of oxidative stress in the present may not simply be the result of decreased lipid accumulation.

Lipid overload in hepatocytes promotes an exacerbation in oxidative stress, which promotes an inflammatory state through release in IL-6 and monocyte chemotactic protein (MCP-1) [33]. Subsequently, there is activation of macrophage infiltration, promoting further release of proinflammatory cytokines [34]. In the present study, we demonstrated that the inflammation of diabetic liver was increased by verifying the increase of CD68, which is a pan-macrophage marker in patients with NASH. Meanwhile, the proinflammatory cytokines such as MCP-1, IL-1*β*, and TNF-*α* were elevated too. Previous studies revealed the anti-inflammation effect of PUR *in vitro* [35, 36]. In the current study, this anti-inflammation effect was embodied in depressing the proinflammatory cytokines and reducing the macrophage infiltrate. The activation of inflammasomes is an important mechanism to initiate inflammation [37]. The NF-*κ*B pathway is crucial in regulating inflammatory stress through activation of TNF signaling and TLRs to increase the transcription of components of the inflammasomes [38]. Cai et al. reported that NF-*κ*B activation is significantly higher in the liver by obesity and high-fat diet [39]. This was also observed in our study. Meanwhile, the key adaptor molecules required for inflammatory processes such as ASC and NLRC4 were also increased. Our results showed that the massive activation of NF-*κ*B and increased ASC and NLRC4 in liver tissue of diabetic liver was downregulated by PUR treatment; this would be the mechanism underlying its inhibition of inflammation in diabetic liver.

Hepatic fibrosis, which is a common result of chronic liver injury, is characterized by the abnormal deposition of extracellular matrix (ECM) proteins [40]. DM increases morbidity and mortality of liver cirrhosis patients [41]. In the present study, diabetic rats showed a significant rise of collagen proteins by picric acid–Sirius red staining and Masson staining. This was mainly due to the injuries of chronic hyperglycemia and lipid disorder in the liver through inflammation and oxidative stress [42]. DM not only accelerates liver inflammation giving rise to more severe liver failure, but may potentiate the incidence of bacterial infections [43]. Inflammatory cytokines play a key role in fibrosis [44]; thus, the persistent inflammation under diabetic condition almost always precedes fibrosis. Sustained hepatic fibrosis can progress to liver cirrhosis, ultimately leading to organ failure and death. PUR exhibited an antifibrotic effect by decreasing the content of collagen types I and III, which are the main components of ECM. Hepatic stellate cells (HSCs) are liver-specific mesenchymal cells and play pivotal roles in liver fibrogenesis. The activation of HSCs is a key event in the development of liver fibrosis. In the current study, we observed high expression of *α*-SMA in diabetic liver, which is the biomarker of activation of HSC. PUR can moderate the activation of HSC evidenced by decreasing *α*-SMA expression.

Activated HSCs also secrete a large amount of TGF-*β*, which in turn stimulates extracellular matrix production and upregulates cell–matrix cell adhesion molecules. Therefore, TGF-*β*1 is considered to be the most important factor in the occurrence and development of liver fibrosis [45]. Thus, in the following work, we mainly focused on the TGF-*β*/Smad signaling pathway to explain the mechanism of PUR suppressing liver fibrosis. After TGF-*β* binding to its receptors T*β*RII and T*β*RI, Smad2/3 was phosphorylated and became activated, then it combined with Smad4 to translocate into the nucleus to initiate the chromatin complex formation and regulate gene expression [46, 47]. In another research, PUR exerted antifibrotic effect through increasing the apoptosis of activated HSC via the downregulation of bcl-2 in the liver tissues [21]. However, this may be a skeptical explanation since apoptosis of hepatocyte is also a stimulator of HSC activation. Nonetheless, activation of HSCs is a complex process involved in many cells including liver sinusoidal endothelial cells besides Kupffer cells and molecules such as ROS, toll-like receptors, and microRNAs. Further work needs to be carried out to elucidate the exact mechanism of the inhibition of PUR on liver fibrosis.

## 5. Conclusion

The present study demonstrated that PUR attenuated liver lipid deposition and liver fibrosis in type 2 diabetic rats by attenuating oxidative stress and inhibiting inflammation. This beneficial effect on diabetic liver injury is related with inhibition on the NF-*κ*B-driven inflammation pathway as well as the TGF-*β*/Smad signaling pathway.

## Figures and Tables

**Table 1:** Sequence of primers used in the present study.

Name		Sequence
MCP-1	Sense (5′-3′)	TCCACCACTATGCAGGTCTC
	Antisense (5′-3′)	GGGCATTAACTGCATCTGGCT
ICAM-1	Sense (5′-3′)	GCCTGGGGTTGGAGACTAAC
	Antisense (5′ -3′)	CTCGCTCTGGGAACGAATACA
VCAM-1	Sense (5′-3′)	GCCACCCTCACCTTAATTGC
	Antisense (5′ -3′)	GAACAACGGAATCCCCAACC
TNF-*α*	Sense (5′-3′)	ATGGGCTCCCTCTCATCAGT
	Antisense (5′ -3′)	GCTTGGTGGTTTGCTACGAC
IL-6	Sense (5′-3′)	TCTCTCCGCAAGAGACTTCCA
	Antisense (5′ -3′)	ATACTGGTCTGTTGTGGGTGG
IL-1*β*	Sense (5′-3′)	GCTTCCTTGTGCAAGTGTCT
	Antisense (5′ -3′)	TCTGGACAGCCCAAGTCAAG
Srebp1c	Sense (5′-3′)	CGTTAACGTGGGTCTCCTCC
	Antisense (5′-3′)	CACTCACCAGGGTCTGCAAG
Scd1	Sense (5′-3′)	GCGTTCCAGAACGATGTGTATG
	Antisense (5′-3′)	CAGAAGCCCAGAACTCAGCTA
Acox	Sense (5′-3′)	AGGAGAAATGGATGCGCCC
	Antisense (5′-3′)	AAGTTTTCCCAAGTCCCCCAG
Cpt1a	Sense (5′-3′)	GTGCAGAGCAATAGGTCCCC
	Antisense (5′-3′)	AGGCAGATCTGTTTGAGGGC
Pepck	Sense (5′-3′)	TGTCCCATTATTGACCCCGC
	Antisense (5′-3′)	ACTTGCCGAAGTTGTAGCCA
G6p	Sense (5′-3′)	TTGTGGTTGGGATACTGGGC
	Antisense (5′-3′)	CTTTTACCCTCGGCCTGGAG
NLRC4	Sense (5′-3′)	TACAGGGACTGATCGGCAGG
	Antisense (5′-3′)	GGGCAGACTGATGTACGCAT
ASC	Sense (5′-3′)	CTGTGCTTAGAGACATGGGCA
	Antisense (5′-3′)	ACAGCTCCAGACTCTTCCATA
GAPDH	Sense (5′-3′)	TACCAGGGCTGCCTTCTCTTG
	Antisense (5′-3′)	GGATCTCGCTCCTGGAAGATG
